# Assessing the causality between thyroid and breast neoplasms: A bidirectional Mendelian randomization study

**DOI:** 10.3389/fonc.2022.973161

**Published:** 2022-12-05

**Authors:** Zhenqi Li, Lu Xia, Xiangzhi Li, Hongyang He

**Affiliations:** ^1^ School of Clinical Medicine, Dali University, Dali, China; ^2^ College of Life Science, Shaanxi Normal University, Xi’an, China; ^3^ Department of General Surgery, The First Affiliated Hospital of Dali University, Dali, China

**Keywords:** thyroid neoplasms, breast neoplasms, Mendelian randomization analysis, database, genetics

## Abstract

**Aim:**

This study aimed to evaluate the association between thyroid neoplasms (TN) and the risk of developing breast neoplasms (BN) by assessing data on single nucleotide polymorphisms (SNPs) obtained from the Deutsches Krebsforschungszentrum (DKFZ) and Breast Cancer Association (BCAC).

**Methods:**

Data on SNPs associated with TN and BN were obtained from DKFZ and BCAC, respectively. Secondary data analysis of all pooled data from genome-wide association studies (GWAS) was performed to identify the genetic loci closely associated with TN or BN as instrumental variables (IVs). To evaluate the causal relationship between TN and BN, a bidirectional Mendelian randomization (MR) analysis was performed using MR Egger regression, weighted median, inverse variance weighted (IVW) random effects model, simple mode, weighted mode, maximum likelihood, penalized weighted median, IVW radial, IVW fixed effects, and robust adjusted profile scores (RAPS) method.

**Results:**

The MR in this study demonstrated a modest reverse causal relationship between TN and BN but a significant positive causal relationship between BN and TN.

**Conclusions:**

The MR of this study provided genetic evidence suggesting an association between BN and TN; however, further research is warranted to explore the potential mechanism of interaction between these two malignancies. Moreover, general breast screening should be performed in individuals with TN, but TN screening should be reinforced in individuals with BN.

## Introduction

Thyroid neoplasms (TN) and breast neoplasms (BN) are the leading causes of cancer in women worldwide. A growing body of research demonstrates a potential two-way pathogenic relationship between BN and TN (1). However, the relationship between the two remains controversial, and the specific mechanism is being extensively evaluated (2). Just as whether radioiodine (RAI) therapy increases cancer risks, this ancient controversy continues (3). However, the breast and thyroid respond to the same endocrine signals. These two malignancies are associated with hormone alteration. Researchers are eager to discuss and examine the association between BN and TN, given the growing number of patients worldwide (4–6). According to a meta-analysis, TN, hyperthyroidism, and autoimmune thyroiditis were significantly associated with an increased risk of BN (7). Mendelian randomization (MR) with two samples established a causal relationship between thyroid dysfunction and BN (8). No previous study has employed MR to investigate a association between these two malignancies. Currently, the primary research approach are observational studies.

Owing to the limitations of conventional statistical methods, observational studies are often hampered by confounding factors and reverse causality, making it challenging to evaluate the observed causation (9). In particular, thyroid and breast diseases are predominant in women, making it difficult to separate these confounding factors. MR is another method that can address these limitations (10). MR eliminates confounding factors by selecting exposure-related genetic variations as instrumental variables (IVs). As alleles are randomly assigned during pregnancy according to Mendel’s second law, it is similar to a natural randomized controlled trial (11). In recent years, while researchers have been satisfied with the study of two samples of MR, they have also begun to utilize two-way MR to explore the two-way relationship between exposures and outcomes, such as the relationship between inflammatory bowel disease and psychiatric diseases (12).

This study applied a bidirectional MR analysis to determine the potential causal relationship between TN and BN.

## Materials and methods

### Data source and study design

**Figure 1:**
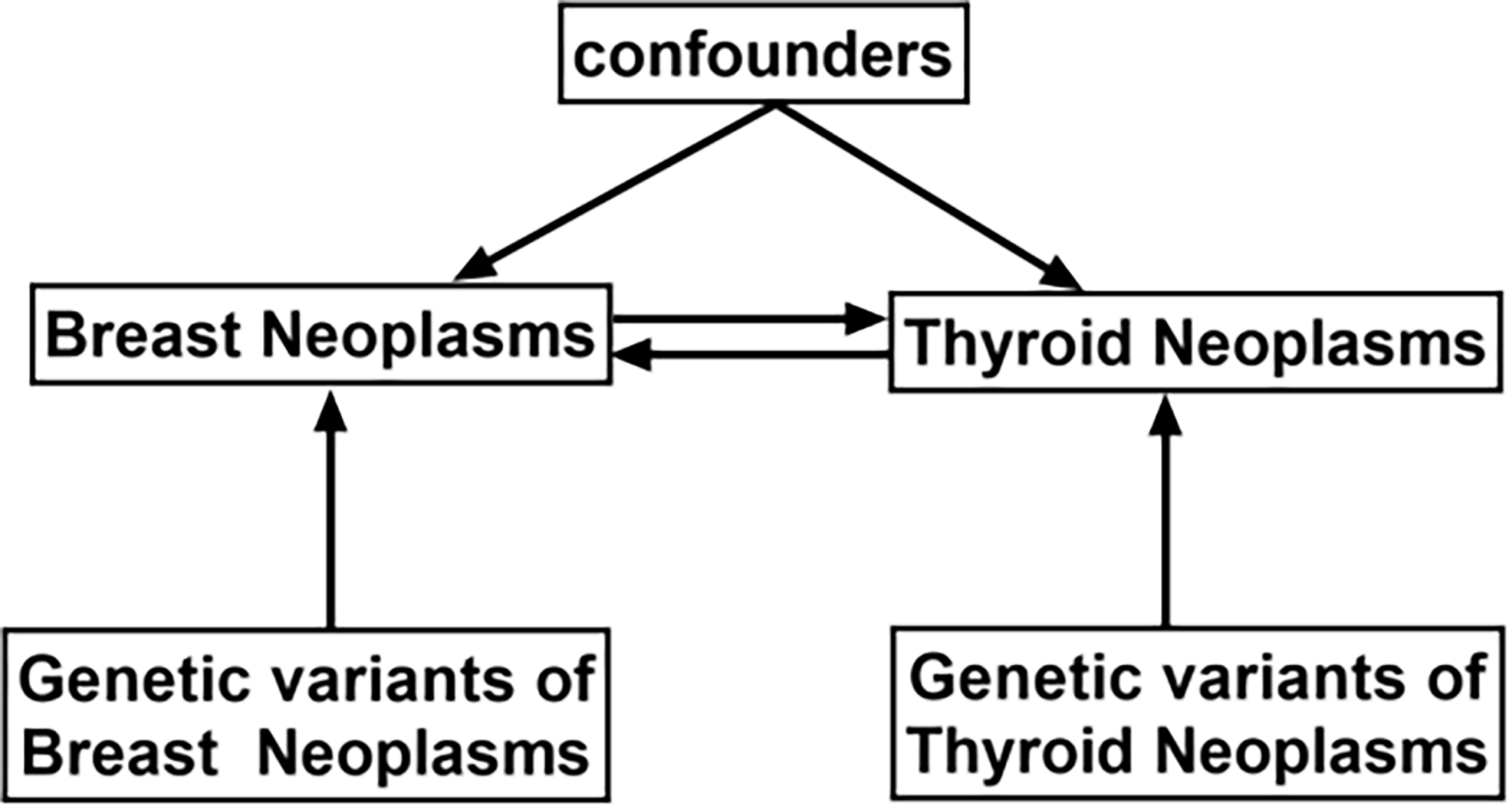
Schematic design showing the study process (hypothesis 1: significantly correlated with exposure; hypothesis 2: not correlated with outcome; hypothesis 3: not correlated with confounders).

The Breast Cancer Association (BCAC) data on variables for genetic variants associated with breast cancer were acquired through genome-wide association studies (GWAS) (https://gwas.mrcieu.ac.uk/), comprising 122,977 breast cancer cases and 105,974 European ancestry controls (13). Similarly, data on thyroid cancer variables were acquired from Deutsches Krebsforschungszentrum (DKFZ) generated using GWAS, which included 649 thyroid cancer cases and 431 European ancestry controls (14). Brief information is shown in **Table 1**. A bidirectional Mendelian randomized MR analysis mode is designed in this study to examine the causative effect of BN and TN (**Figure 1**).

**Table 1:** Summary of the GWAS included in this MR study neoplasms.

Exposures/outcomes	GWASID	Consortium	Ethnicity	Sample sizes	Number of SNPs	Sex	Year
Thyroid cancer	ieu-a-1082	DKFZ	European	1,080	572,028	Male and Female	2013
Breast cancer	ieu-a-1126	BCAC	European	228,951	10,680,257	Female	2017

GWAS, genome-wide association studies; SNPs, single nucleotide polymorphisms; IVs, instrumental variables; DKFZ, Deutsches Krebsforschungszentrum; BCAC, Breast Cancer Association.

### Selection of genetic instrumental variables

The genetic variants of single nucleotide polymorphisms (SNPs) with genome-wide significance (*p*<5×10^−8^) in BN/TN were selected for pooling. To select the relevant SNPs, the threshold value of the linkage disequilibrium parameter (*r^2^*) was set to 0.001, whereas the genetic distance was set to 10,000 kb to ensure independence and rule out the effect of linkage disequilibrium on the results. SNPs whose corresponding phenotypes were connected with selected outcome variables were eliminated, leaving only those SNPs that fulfilled hypotheses 1, 2, and 3. GWAS was utilized to extract information on the outcome of BN/TN and to determine the relationship between SNPs and outcomes that satisfied hypotheses 1, 2, and 3. Then, the exposure and outcome datasets were merged. This integrated dataset comprised the above instrumental variables alongside the outcome and exposure, and the echo SNPs were removed. The remaining SNPs were considered as instrumental variables.

### Statistical analysis

The MR-Egger regression method (15), median weighting method (16), inverse variance weighted (IVW) random effects model (17), simple mode, weighted method (16), maximum likelihood ratio method (18), linear median weighted method (16), IVW radial method (19), IVW fixed effects model (17), and RAPS method were applied to perform a two-sample MR analysis. In addition, the odds ratios and 95% confidence intervals (CIs) for the 10 approaches were presented. Among them, the inverse variance weighting approach, which does not consider the existence of an intercept term in regression and employs the inverse of result variance (standard error quadratic) as the weight for fitting, was used in the IVW random effects model and IVW fixed effects model. The Wald ratio method was implemented to quantify the exposure-outcome impact of each SNP, followed by the weighted linear regression with a forced intercept of zero. When the IVs fulfill the three principal assumptions, it improves the estimation accuracy and testing capabilities. However, the MR-Egger regression method considers the presence of an intercept term. When pleiotropy occurs in IVs, the estimation of the causal effects is skewed. The MR approach using multiple instrumental variables on the basis of the summary data adapted according to IVW is known as MR-Egger regression. Unlike IVW, this method only needs to satisfy the assumption that the pleiotropic effect of instrumental variables is independent of the association between IVS and exposure factors, as well as the assumption of no measurement error, which is less stringent than the three core assumptions of instrumental variables. This approach can identify pleiotropy in addition to rectifying the pleiotropy bias. Therefore, the MR-Egger regression can maintain the validity of the MR method in studies with multiple genetic variants considered as instrumental variables. The RAPS method, which does not consider horizontal pleiotropy or outliers, is a relatively recent approach. In international MR analysis, two approaches (IVW and MR-Egger) are often used as fundamental MR methods, whereas the other seven methods are innovative MR analysis approaches introduced in recent years. In this study, the results of IVW were supplemented and demonstrated as the main results of the analysis.

Cochran’s Q test was used to assess the heterogeneity of the individual genetic variance estimates. If the *p value* was <0.05 for Cochran’s Q test, the results of MR were referred to as the IVW multiplicative random effects; otherwise, the IVW fixed effects were used to visualize the results of heterogeneity test with a forest plot. The Egger-intercept technique of horizontal pleiotropy was used to determine any violations of the MR hypothesis due to horizontal pleiotropy, with the truncation value suggesting the degree of influence of genetic variations on the outcome through a pathway other than exposure. Horizontal pleiotropy should also be assessed by observing for asymmetry in the funnel plot to measure the reliability of the current MR analysis using the leave-one-out method and to determine whether any of the three SNPs were outliers. The two-sample MR analysis findings were complemented with new approaches including the maximum likelihood method, penalized weighted median, IVW radial method, and IVW fixed effects. Gold standard test results were obtained using the IVW technique. Finally, the radio package was employed to display the data and to detect any genuine outliers. If outliers were observed, the MRPRESSO approach was utilized to examine and to discuss the potential impact on the results.

## Results

The remaining 347 SNPs of thyroid cancer-related genetic variation that simultaneously meet assumptions 1, 2, and 3 were screened from the human genotype-phenotype association database; meanwhile, the SNPs related to smoking (rs6546667, rs7849585, rs12441088, and rs2157787), hyperlipidemia (rs4567782), hypercholesterolemia (rs11692610), menopausal age (rs10031777), number of live births (rs12651136), menarche age (rs1077420), alcohol (rs461599 and rs6060124), and obesity (rs2483374) were deleted. As the aforementioned 12 SNPs were removed, only 335 were retained. Information on breast cancer was extracted by GWAS, and the relationship between the above 335 SNPs and the outcome was determined by analyzing the study outcomes. As the BCAC were unable to determine 19 RS loci in breast cancer, 316 SNPs remained. Hence, the exposure and outcome dataset were merged, which showed the relationship between the 316 tool variables and the outcomes and exposures, and palindrome SNPs were deleted (n=0). The 316 instrumental variables were considered as the final instrumental variables referring to thyroid cancer. The remaining 142 SNPs of breast cancer-related genetic variations that simultaneously met hypotheses 1, 2, and 3 were screened from the human genotype-phenotype association database, and those related to abnormal nail function (rs1230666 and rs1121948), smoking (rs11205303, rs73949122, and rs11672660), thyroid carcinoma (rs12990503), and diabetes (rs10885405, rs62048402, and rs56013747) were deleted. Hence, 133 SNPs remained. Information about the outcome of thyroid cancer was extracted by GWAS, and the relationship between the 133 SNPs and the outcome was determined by analyzing the study outcomes. As DKFZ did not determine 112 RS loci in breast cancer, 21 SNPs were retained. The exposure and outcome dataset were merged, which showed the relationship between the above 21 tool variables and the outcomes and exposures, and the palindrome SNPs were deleted (n=0). The last 21 instrumental variables were assigned as the final instrumental variables referring to the breast.

**Figure 2:**
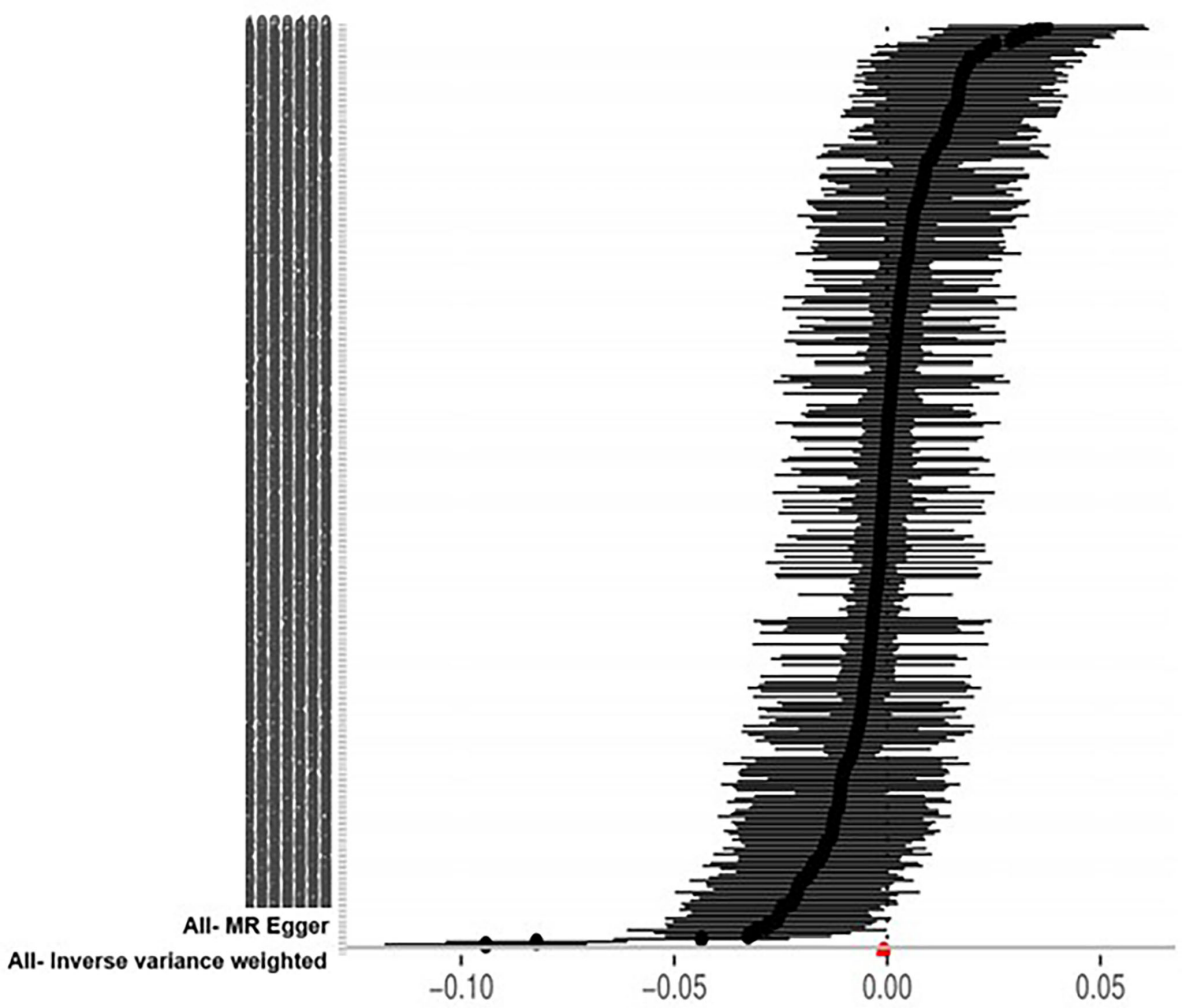
Forest diagram of instrumental variable (TN→BN).

**Figure 3:**
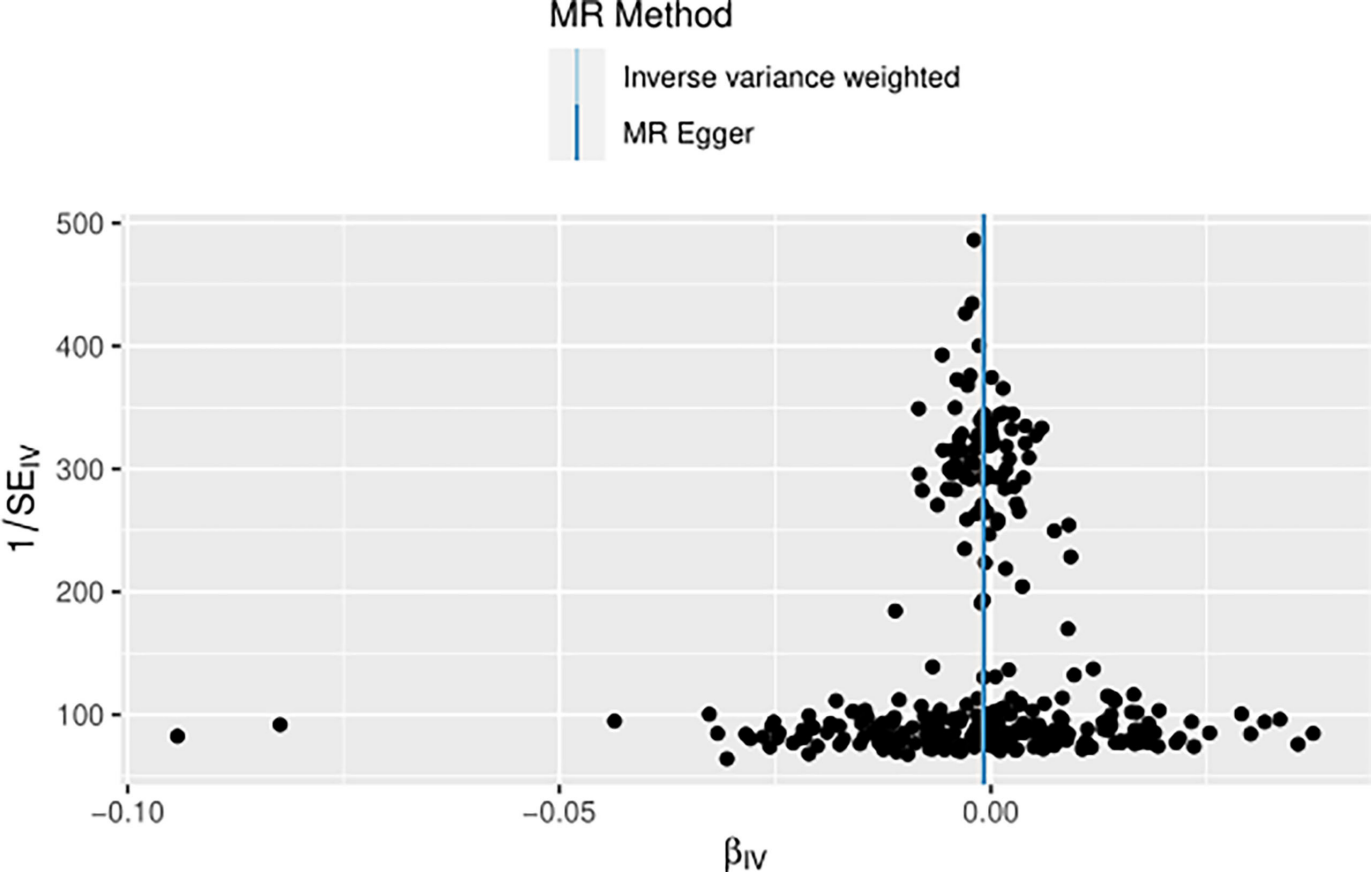
Funnel diagram of instrumental variable (TN→BN).

**Figure 4:**
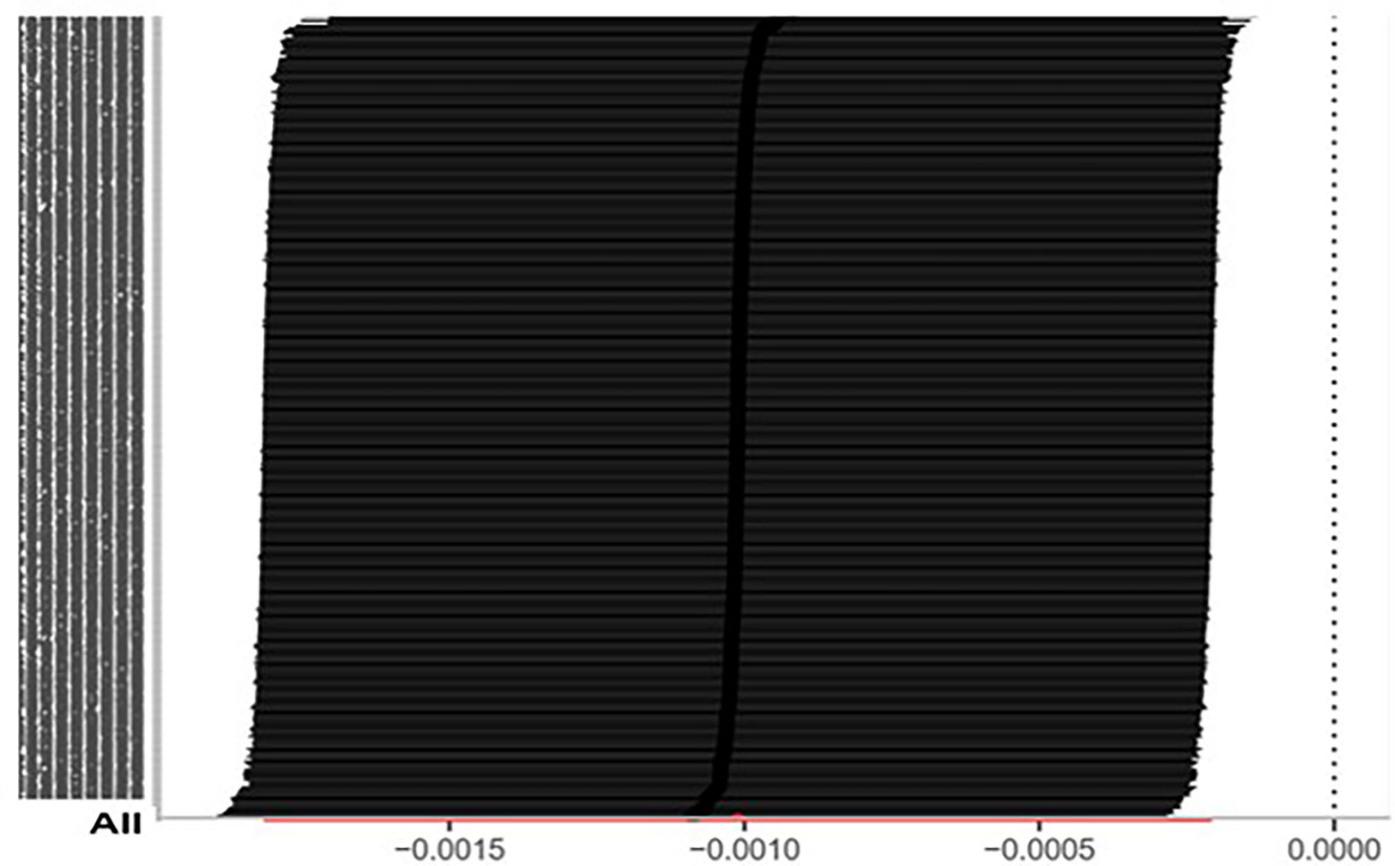
Inspection chart of “leave one method” (TN→BN).

The IVW approach computed the heterogeneity of MR results from thyroid cancer to breast cancer (*p*=2.1133e-13), showing obvious heterogeneity; however, the funnel diagram exhibited greater symmetry, suggesting that the results were stable. The results of the level pleiotropy test were obtained using the Egger’s intercept method (*p*=0.7008), which showed that the instrumental variables did not affect the outcome (BN) through ways other than exposure (TN). The results of the left-hand method were very stable. The forest diagram of instrumental variables illustrates the funnel diagram and forest plot of the results of the leave-one-out method (**Figures 2**–**4**).

**Figure 5:**
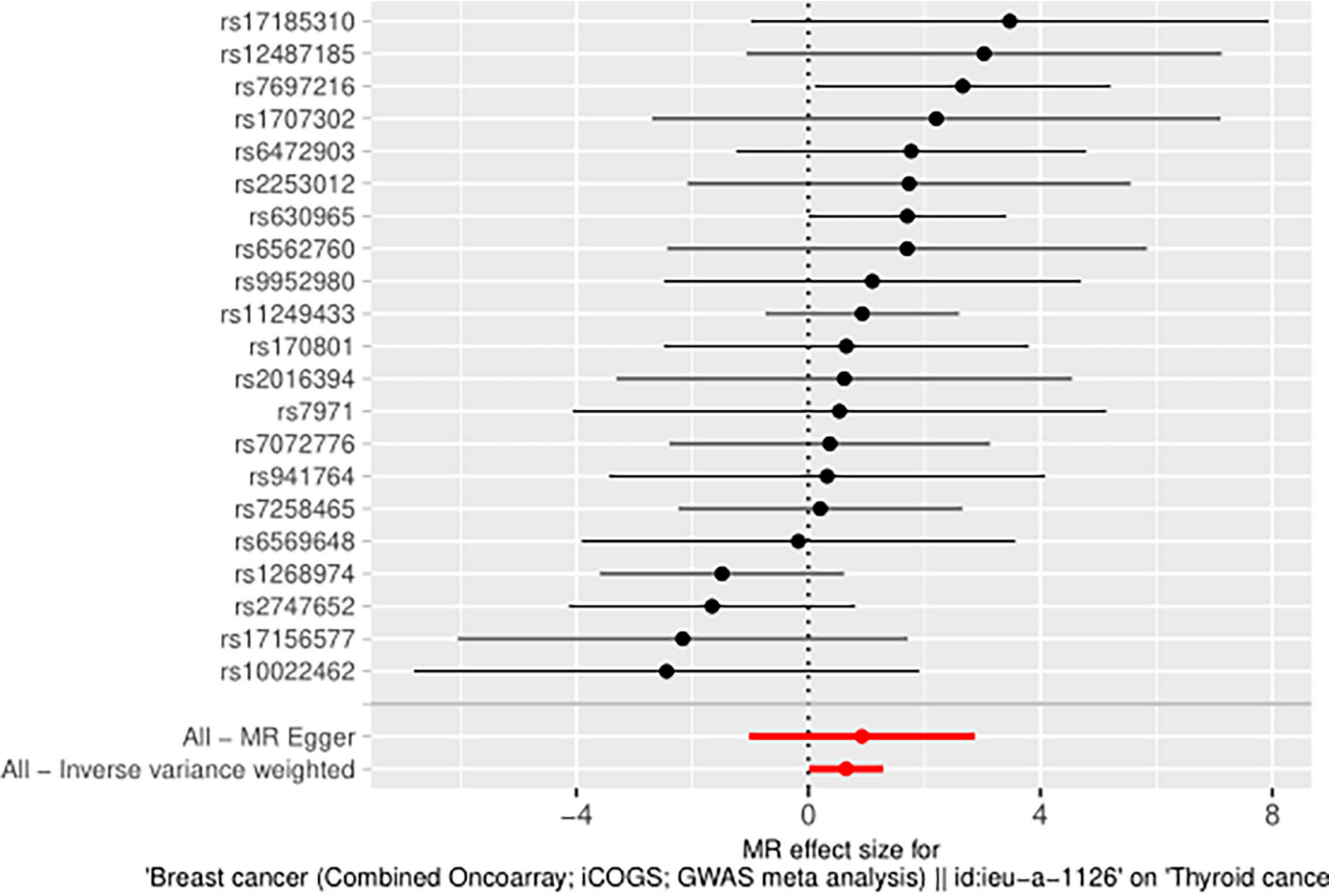
Forest diagram of instrumental variable (BN→TN).

**Figure 6:**
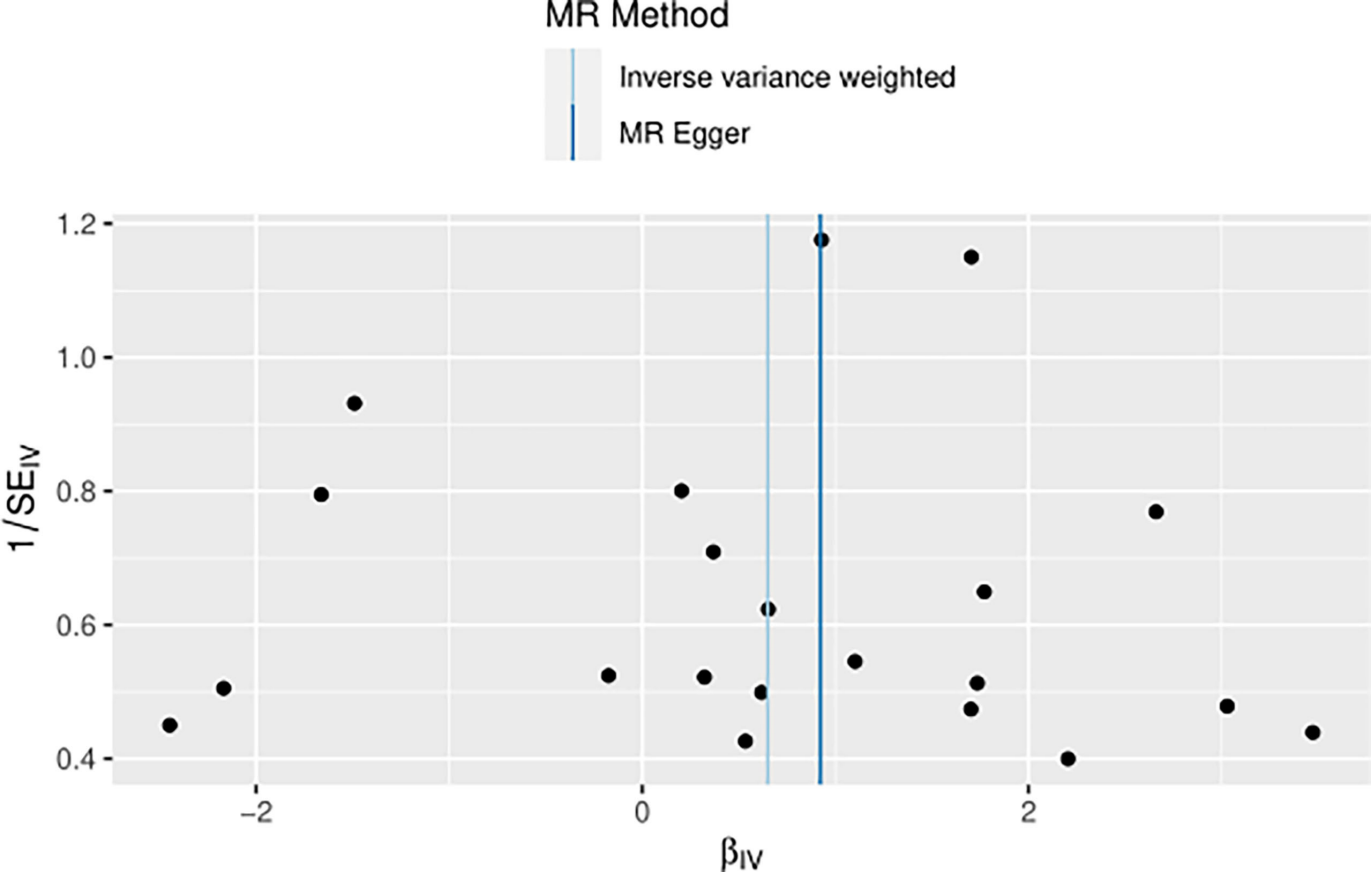
Funnel diagram of instrumental variable (BN→TN).

**Figure 7:**
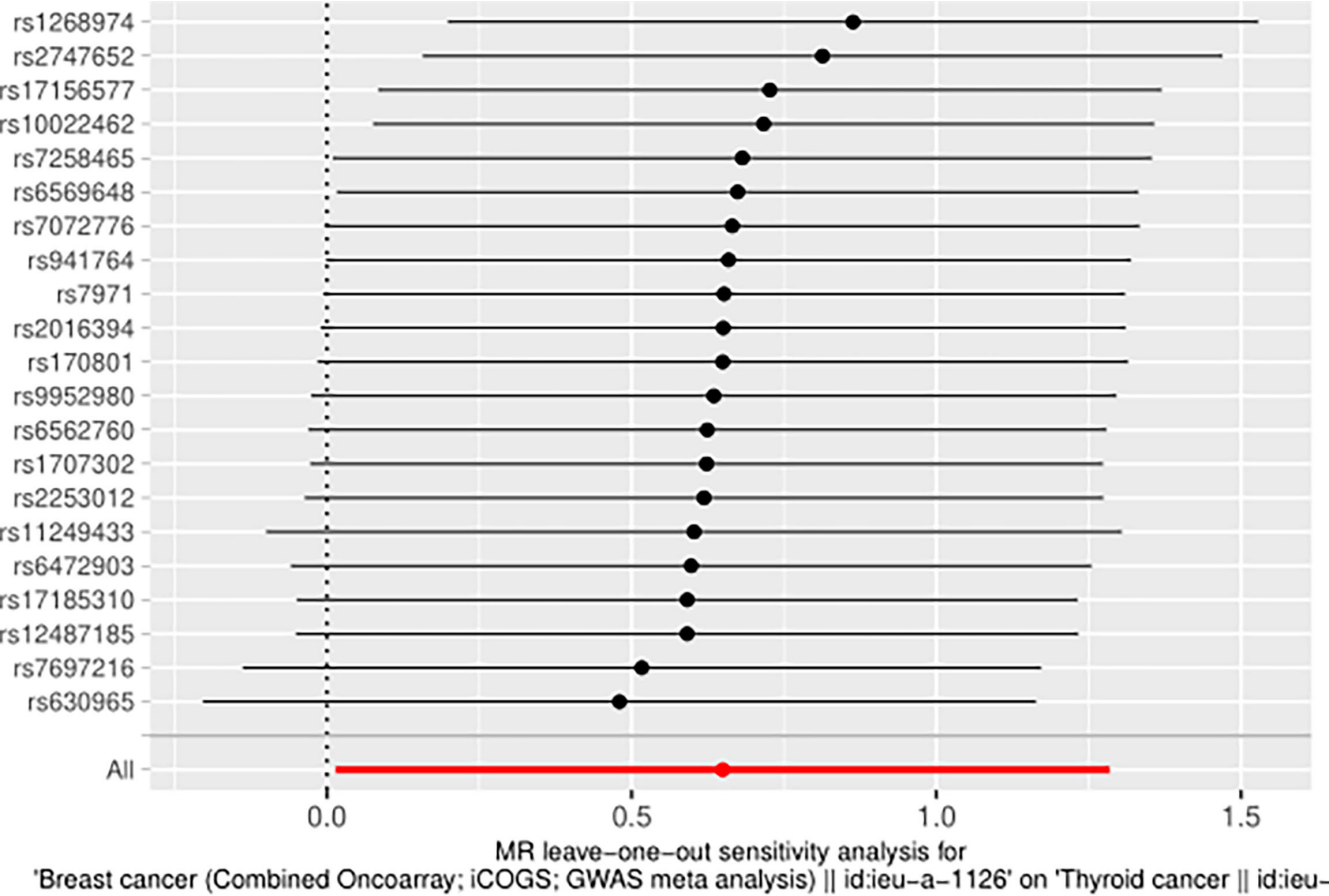
Inspection chart of “leave one method” (BN→TN).

The heterogeneity of MR results from breast cancer to thyroid cancer calculated using the IVW method was *p*=0.4551, indicating the absence of noticeable heterogeneity; however, the funnel diagram was asymmetric. The Egger’s intercept approach yielded level pleiotropy test findings (*p*=0.7736), thus indicating that the instrumental variables did not affect the outcome (thyroid cancer) in ways other than exposure (breast cancer). The results of the left-hand method were very stable. The forest diagram of instrumental variables shows the funnel diagram and forest diagram of the results of the leave one method (**Figures 5**–**7**).

**Figure 8:**
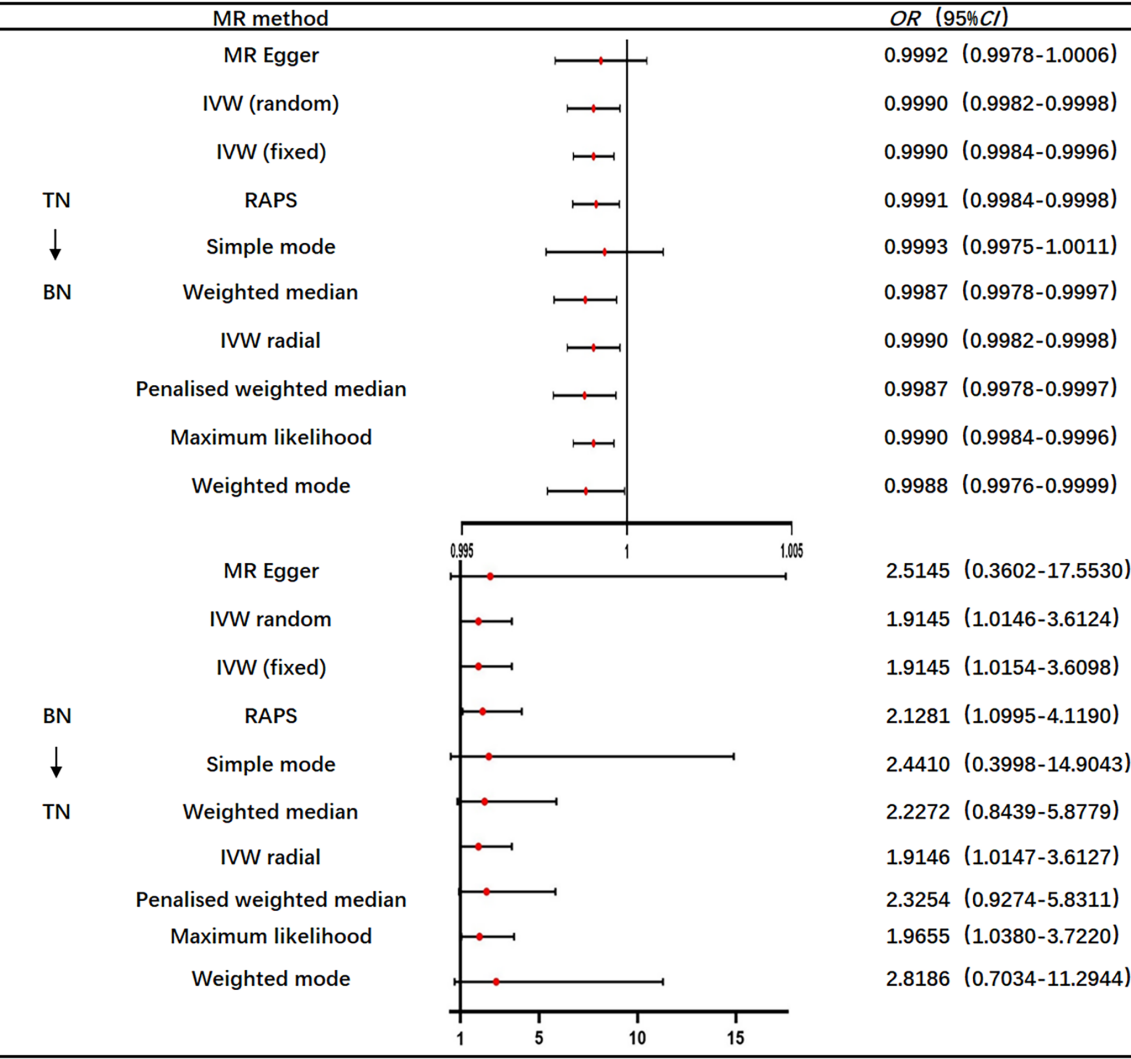
OR value of the 10 methods (TN→BN; BN→TN).

Ten approaches were applied to augment the results of the MR analysis. As can be seen, TN was the exposure variable, while BN was the outcome variable. The simple mode and MR-Egger regression method OR values and 95% CIs were not significant. Due to the presence of heterogeneity, the IVW random effects model was used as the gold standard (n=316snps, OR=0.9990, 95% CI: 0.9982–0.9998, *p*=1.36e-02). A correlation was observed between the median weighting method, IVW random effects model, weighting method, maximum likelihood ratio, linear median weighting method, IVW radial method, and IVW fixed effects model. This finding is almost consistent with that of the RAPS method, which shows that TN has a negative causal relationship with BN (**Figure 8**).

Taking BN as the exposure variable and thyroid cancer as the outcome variable, the OR values and 95% CIs calculated using the MR-Egger regression method, median weighted method, simple mode, weighted method, and linear median weighted method were not considered significant. Due to the absence of heterogeneity, the IVW fixed effects model was used as the gold standard (n=21snps, OR=1.9146, 95% CI: 1.0147–3.6127, *p*=4.47e-02). The associations observed among the results of IVW random effects model, maximum likelihood ratio, IVW radial method, and RAPS method are similar, which shows that BN has a positive causal relationship with TN (**Figure 8**).

**Figure 9:**
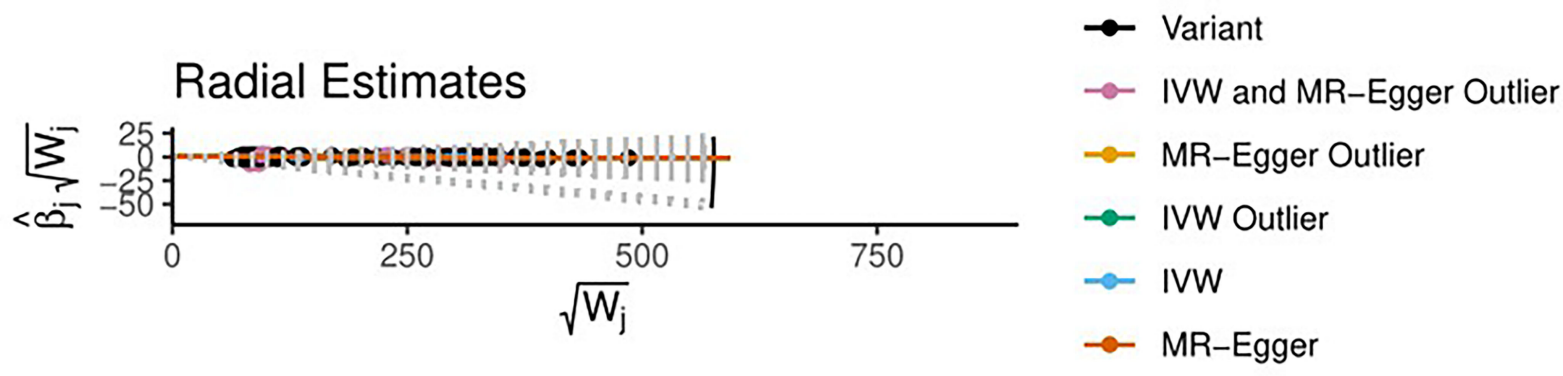
Diagram of the radial estimates (TN→BN).

**Figure 10:**
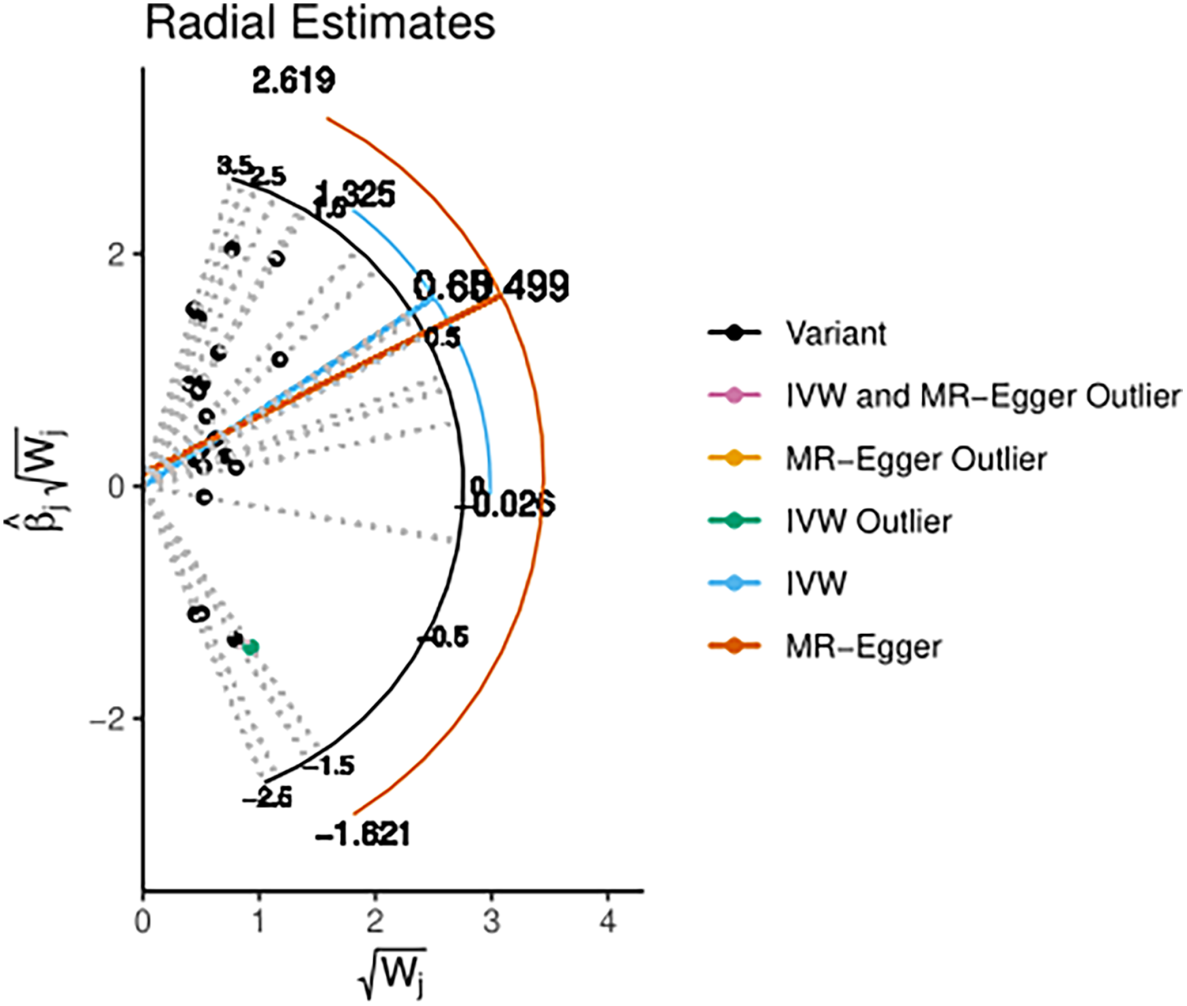
Diagram of the radial estimates (BN→TN).

Using radio to identify the outliers, it is feasible to demonstrate that if thyroid cancer is the exposure variable and breast cancer variable is the outcome variable, there are four outliers among the 316 tool variables screened. After using the MRPRESSO method for verification, the initial *p value* was 0.0004; after correction and elimination of the four outliers, the *p value* was 0.0004. The outliers had a minimal impact on the results, demonstrating that the study findings are resilient. However, with breast cancer as the exposure variable and thyroid cancer as the outcome variable, no outliers were found in the selected 21 instrumental variables (**Figures 9**, **10**).

Finally, the overall *R* ^2^ value (0.6785) of 21 tool variables referring to breast cancer and the *F* value (106.3111) were estimated using the *R* ^2^ and F value calculation algorithm, respectively; results showed that 21 tool variables related to BN were strong tool variables. The overall *R* ^2^ value of the 316 thyroid cancer tool variables was 0.0687, while the *F* value was 53.3341, illustrating that the 316 tool variables chosen for this study were also strong tool variables.

## Discussion

In this bidirectional MR study, a weak reverse causal relationship was observed between TN and BN, but a strong positive causal relationship was found between BN and TN.

BN and TN are two malignant tumors with the highest incidence rates in women, and data demonstrating a reciprocal positive causal relationship between the two continue to emerge. Some researchers believe that this is due not only to genetic variations but also to the environment and the respective chemoradiotherapy used; thus, the causal relationship between the two is affected by numerous confounding factors (20). This study serves to eradicate the confounding factors due to genetic variations. However, in terms of genetic variation, the findings of this study are consistent with those of most studies; that is, BN patients are more likely to develop TN, while TN is a predisposing factor for developing BN. Surprisingly, Van et al. evaluated the data from the American Cancer Society and found that the incidence of TN in female patients with BN nearly tripled in 2013 (21). Taking the IVW fixed effects model as an example, our MR OR value (1.9146, 95% CI: 1.0147–3.6127) undoubtedly coincides with this finding. In a multicenter study conducted this year, the presence or absence of BN was not linked to the presence or absence of TN (22). These observational research results are contradictory and are difficult to prove. However, most studies have recognized a mutually pathogenic relationship between the two malignancies. Although our OR value from TN to BN showed an extremely small negative causal relationship, we cannot explain the underlying reason for this difference (23). However, a very interesting study by Lei et al. showed that patients with secondary metachronous TN have a relatively good prognosis for BN (24). This study validated our preliminary conclusions. TN may be a protective factor against BN and may also lead to a better prognosis in BN patients who will develop TN in the future. A previous study conducted in 400,000 individuals also found that patients who developed two types of cancer had a low invasive BN tumor behavior. Another previous study using the SEER database obtained the same conclusion (25, 26). A study of 13,978 BN patients showed that the prognosis of both isochronous and synchronous cancers was worse than that of a single cancer, which seems rational; however, several confounding factors were noted in the definition of isochronous and synchronous cancers (27). In conclusion, the causal relationship between BN and TN in our MR study was consistent with the findings of most related studies, and the reverse causal relationship seems unimaginable. However, our results are robust, although they are considered weak. According to several studies, these diseases are related to the high prevalence of SNP. This study relied on genetic variation points (28). Moreover, although parp4 and CHEK2 mutations are important markers (1, 29), the association between certain classic markers, such as thyroid autoantibodies, thyroid hormone receptors α and β, estrogen receptor α, and breast and thyroid cancers requires further exploration (30–32).

An extremely weak reverse causality was observed between TN and BN, while a strong positive causality was found between BN and TN. From an epidemiological point of view, screening for both malignancies is necessary (33). The following recommendations were proposed based on the MR results: individuals with thyroid cancer should undergo general breast screening. The person in charge should reassure the patients regarding the necessity of the screening to reduce their anxiety. However, thyroid color ultrasonography and thyroid function examination should be employed to enhance TN screening in patients with BN. This may guide clinicians in making appropriate treatment decisions and increase the patients’ awareness of the need to undergo physical examinations. Moreover, a cross-sectional study from China based on ultrasound screening showed that breast masses and thyroid nodules are susceptible to common diseases, regardless of whether the test results are negative or positive (34), complicating the relationship between breast nails and highlighting the importance of co-screening breast nails.

This study has several limitations. First, the number of patients with TN in the study was relatively small due to dataset constraints, and the genetic data were collected from ordinary women, which may have led to bias. Second, our dataset only included European populations, limiting the application of the conclusions to non-European groups. Hence, further studies are warranted to verify the applicability of these results to different populations and races. Third, our reverse causal inquiry revealed heterogeneity and unstable results, although the final results were robust.

However, the MR in this study demonstrated a causal relationship between thyroid and breast cancers. Although the specific mechanism needs to be explored further, it provides evidence for the rationality of screening some cancer patients.

## Conclusion

Overall, this study established a causal relationship between TN and BN. Future studies should involve people from other demographics and explore the potential mechanisms of action between the variables. At the same time, general breast screening should be performed in individuals with TN, but TN screening should be reinforced in individuals with BN.

## Data availability statement

The original contributions presented in the study are included in the article/**Supplementary Material**. Further inquiries can be directed to the corresponding author.

## Ethics statement

Ethical review and approval was not required for the study on human participants in accordance with the local legislation and institutional requirements. Written informed consent for participation was not required for this study in accordance with the national legislation and the institutional requirements.

## Author contributions

ZL wrote the manuscript and performed the quality assessment. LX designed the project and performed the statistical analysis. LX has the same contribution as ZL. XL and HH contributed to the revision of the manuscript and reviewed the results. All authors contributed to the article and approved the submitted version.

## Acknowledgments

We would like to thank HH for providing helpful advice regarding the methodology and statistics. We would like to thank Editage (www.editage.cn) for English language editing.

## Conflict of interest

The authors declare that the research was conducted in the absence of any commercial or financial relationships that could be construed as a potential conflict of interest.

## Publisher’s note

All claims expressed in this article are solely those of the authors and do not necessarily represent those of their affiliated organizations, or those of the publisher, the editors and the reviewers. Any product that may be evaluated in this article, or claim that may be made by its manufacturer, is not guaranteed or endorsed by the publisher.

## Abbreviations

TN, thyroid neoplasms; BN, breast neoplasms; SNPs, single nucleotide polymorphisms; IVs, instrumental variables; DKFZ, Deutsches Krebsforschungszentrum; BCAC, Breast Cancer Association.

## References

[1] Ikeda Y Kiyotani K Yew PY Kato T Tamura K Yap KL. Germline PARP4 mutations in patients with primary thyroid and breast cancers. Endocr Relat Cancer (2016) 23(3):171–9. doi: 10.1530/erc-15-0359 PMC5152685 26699384

[2] Baldini E Lauro A Tripodi D Pironi D Amabile MI Ferent IC. Thyroid diseases and breast cancer. J Pers Med (2022) 12(2). doi: 10.3390/jpm12020156 PMC8876618 35207645

[3] Shim SR Kitahara CM Cha ES Kim SJ Bang YJ Lee WJ. Cancer risk after radioactive iodine treatment for hyperthyroidism: A systematic review and meta-analysis. JAMA Netw Open (2021) 4(9):e2125072. doi: 10.1001/jamanetworkopen.2021.25072 34533571 PMC8449277

[4] Fei X Christakos G Lou Z Ren Y Liu Q Wu J. Spatiotemporal Co-existence of female thyroid and breast cancers in hangzhou, China. Sci Rep (2016) 6:28524. doi: 10.1038/srep28524 27341638 PMC4920092

[5] Joseph KR Edirimanne S Eslick GD. The association between breast cancer and thyroid cancer: A meta-analysis. Breast Cancer Res Treat (2015) 152(1):173–81. doi: 10.1007/s10549-015-3456-6 26058757

[6] An JH Hwangbo Y Ahn HY Keam B Lee KE Han W. A possible association between thyroid cancer and breast cancer. Thyroid (2015) 25(12):1330–8. doi: 10.1089/thy.2014.0561 26442580

[7] Chen S Wu F Hai R You Q Xie L Shu L. Thyroid disease is associated with an increased risk of breast cancer: A systematic review and meta-analysis. Gland Surg (2021) 10(1):336–46. doi: 10.21037/gs-20-878 PMC7882351 33633990

[8] Yuan S Kar S Vithayathil M Carter P Mason AM Burgess S. Causal associations of thyroid function and dysfunction with overall, breast and thyroid cancer: A two-sample mendelian randomization study. Int J Cancer (2020) 147(7):1895–903. doi: 10.1002/ijc.32988 PMC7611568 32215913

[9] Del Rio P Viani L Bonati E Marina M Arcuri MF Ceresini G. Possible association between breast thyroid carcinoma: analysis of risk factors. Ann Ital Chir (2020) 91:173–80. 32719185

[10] Davey Smith G Hemani G. Mendelian randomization: genetic anchors for causal inference in epidemiological studies. Hum Mol Genet (2014) 23(R1):R89–98. doi: 10.1093/hmg/ddu328 PMC4170722 25064373

[11] Emdin CA Khera AV Kathiresan S. Mendelian randomization. Jama (2017) 318(19):1925–6. doi: 10.1001/jama.2017.17219 29164242

[12] Luo J Xu Z Noordam R Van Heemst D Li-Gao R. Depression and inflammatory bowel disease: A bidirectional two-sample mendelian randomization study. J Crohns Colitis (2022) 16(4):633–42. doi: 10.1093/ecco-jcc/jjab191 34739073

[13] Michailidou K Lindström S Dennis J Beesley J Hui S Kar S. Association analysis identifies 65 new breast cancer risk loci. Nature (2017) 551(7678):92–4. doi: 10.1038/nature24284 PMC5798588 29059683

[14] Köhler A Chen B Gemignani F Elisei R Romei C Figlioli G. Genome-wide association study on differentiated thyroid cancer. J Clin Endocrinol Metab (2013) 98(10):E1674–81. doi: 10.1210/jc.2013-1941 23894154

[15] Bowden J Davey Smith G Burgess S. Mendelian randomization with invalid instruments: effect estimation and bias detection through egger regression. Int J Epidemiol (2015) 44(2):512–25. doi: 10.1093/ije/dyv080 PMC4469799 26050253

[16] Bowden J Davey Smith G Haycock PC Burgess S. Consistent estimation in mendelian randomization with some invalid instruments using a weighted median estimator. Genet Epidemiol (2016) 40(4):304–14. doi: 10.1002/gepi.21965 PMC4849733 27061298

[17] Burgess S Butterworth A Thompson SG. Mendelian randomization analysis with multiple genetic variants using summarized data. Genet Epidemiol (2013) 37(7):658–65. doi: 10.1002/gepi.21758 PMC4377079 24114802

[18] Davies NM Von Hinke Kessler Scholder S Farbmacher H Burgess S Windmeijer F Smith GD. The many weak instruments problem and mendelian randomization. Stat Med (2015) 34(3):454–68. doi: 10.1002/sim.6358 PMC4305205 25382280

[19] Bowden J Spiller W Del Greco MF Sheehan N Thompson J Minelli C. Improving the visualization, interpretation and analysis of two-sample summary data mendelian randomization *via* the radial plot and radial regression. Int J Epidemiol (2018) 47(4):1264–78. doi: 10.1093/ije/dyy101 PMC6124632 29961852

[20] Bolf EL Sprague BL Carr FE. A linkage between thyroid and breast cancer: A common etiology? Cancer Epidemiol Biomarkers Prev (2019) 28(4):643–9. doi: 10.1158/1055-9965.Epi-18-0877 30541751

[21] Van Fossen VL Wilhelm SM Eaton JL Mchenry CR. Association of thyroid, breast and renal cell cancer: A population-based study of the prevalence of second malignancies. Ann Surg Oncol (2013) 20(4):1341–7. doi: 10.1245/s10434-012-2718-3 23263698

[22] Peckham M Spencer HJ Syed S Armstrong WB Farwell DG Gal TJ. Breast and thyroid cancer: A multicenter study with accrual to clinical trials network. J Surg Oncol (2022) 125(8):1211–7. doi: 10.1002/jso.26825 PMC9106860 35195923

[23] Jin YJ Kwon MJ Kim JH Kim JH Choi HG. Association between thyroid cancer and breast cancer: Two longitudinal follow-up studies using a national health screening cohort. J Pers Med (2022) 12(2). doi: 10.3390/jpm12020133 PMC8880453 35207622

[24] Lei K He X Yu L Ni C Chen H Guan D. Breast cancer prognosis is better in patients who develop subsequent metachronous thyroid cancer. PloS One (2019) 14(5):e0215948. doi: 10.1371/journal.pone.0215948 31042767 PMC6493754

[25] Cheng W Shen X Xing M. Decreased breast cancer-specific mortality risk in patients with a history of thyroid cancer. PloS One (2019) 14(10):e0221093. doi: 10.1371/journal.pone.0221093 31644578 PMC6808426

[26] Li S Yang J Shen Y Zhao X Zhang L Wang B. Clinicopathological features, survival and risk in breast cancer survivors with thyroid cancer: An analysis of the SEER database. BMC Public Health (2019) 19(1):1592. doi: 10.1186/s12889-019-7947-y 31783815 PMC6884836

[27] Huang NS Chen XX Wei WJ Mo M Chen JY Ma B. Association between breast cancer and thyroid cancer: A study based on 13 978 patients with breast cancer. Cancer Med (2018) 7(12):6393–400. doi: 10.1002/cam4.1856 PMC6308067 30480382

[28] Bakos B Kiss A Árvai K Szili B Deák-Kocsis B Tobiás B. Co-Occurrence of thyroid and breast cancer is associated with an increased oncogenic SNP burden. BMC Cancer (2021) 21(1):706. doi: 10.1186/s12885-021-08377-4 34130653 PMC8207626

[29] Cieszyńska M Kluźniak W Wokołorczyk D Cybulski C Huzarski T Gronwald J. Risk of second primary thyroid cancer in women with breast cancer. Cancers (Basel) (2022) 14(4). doi: 10.3390/cancers14040957 PMC8870271 35205705

[30] Prinzi N Baldini E Sorrenti S De Vito C Tuccilli C Catania A. Prevalence of breast cancer in thyroid diseases: results of a cross-sectional study of 3,921 patients. Breast Cancer Res Treat (2014) 144(3):683–8. doi: 10.1007/s10549-014-2893-y 24604093

[31] Kim YA Kim YA Cho SW Song YS Min HS Park IA. Increased expression of thyroid hormone receptor alpha and estrogen receptor alpha in breast cancer associated with thyroid cancer. Eur J Surg Oncol (2021) 47(6):1316–23. doi: 10.1016/j.ejso.2021.01.015 33558123

[32] Bolf EL Gillis NE Davidson CD Cozzens LM Kogut S Tomczak JA. Common tumor-suppressive signaling of thyroid hormone receptor beta in breast and thyroid cancer cells. Mol Carcinog (2021) 60(12):874–85. doi: 10.1002/mc.23352 PMC8585704 34534367

[33] Kuo JH Chabot JA Lee JA. Breast cancer in thyroid cancer survivors: An analysis of the surveillance, epidemiology, and end results-9 database. Surgery (2016) 159(1):23–9. doi: 10.1016/j.surg.2015.10.009 26522696

[34] Li H Wang Z Liu JS Zou BS Chen HR Xu Z. Association between breast and thyroid lesions: A cross-sectional study based on ultrasonography screening in China. Thyroid (2020) 30(8):1150–8. doi: 10.1089/thy.2019.0184 32148169

