# Supplementary figures and images for: Assessing the causality between thyroid and breast neoplasms: A bidirectional Mendelian randomization study
Source: Front Oncol. 2022 Dec 5;12:973161. doi: 10.3389/fonc.2022.973161 (PMC9760946; doi:10.3389/fonc.2022.973161)

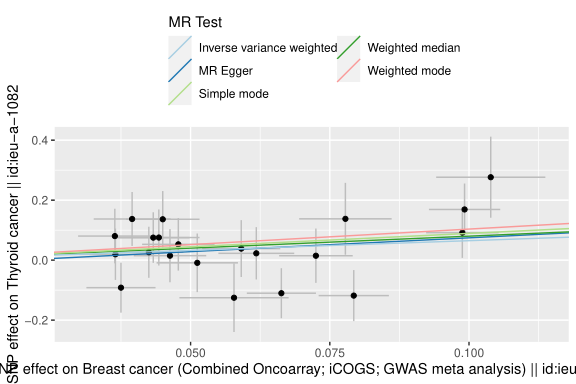

Supplement: Supplementary file 5 [file Image_1.jpeg]

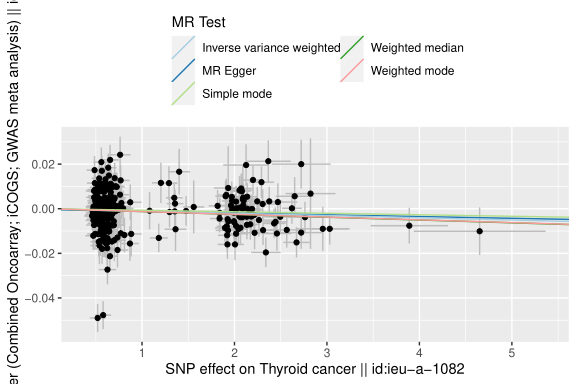

Supplement: Supplementary file 6 [file Image_2.jpeg]
